# High-Speed Infrared Radiation Thermometer for the Investigation of Early Stage Explosive Development and Fireball Expansion

**DOI:** 10.3390/s22166143

**Published:** 2022-08-17

**Authors:** Matthew J. Hobbs, Andrew Barr, Scott Woolford, Dain Farrimond, Sam D. Clarke, Andrew Tyas, Jon R. Willmott

**Affiliations:** 1 Sensor Systems Group, Electronic & Electrical Engineering Department, University of Sheffield, Portobello Centre, Pitt Street, Sheffield S1 4ET, UK; 2 Department of Civil & Structural Engineering, University of Sheffield, Sir Frederick Mappin Building, Mappin Street, Sheffield S1 3JD, UK

**Keywords:** radiation thermometry, infrared radiation thermometer, blast loading, confined blast, temperature measurements, fireball, afterburn

## Abstract

The understanding of blast loads is critical for the development of infrastructure that protects against explosions. However, the lack of high-quality experimental work on the characterisation of such loads prevents a better understanding of many scenarios. Blast loads are typically characterised by use of some form of pressure gauge, from which the temperature can be inferred from a pressure measurement. However, such an approach to temperature measurement is limited; it assumes ideal gas laws apply throughout, which may not be the case for high temperature and pressure scenarios. In contrast, infrared radiation thermometers (IRTs) perform a measurement of temperature based upon the emitted radiance from the target object. The IRTs can measure fast changes in transient temperature, making them seemingly ideal for the measurement of a fireball’s temperature. In this work, we present the use of a high-speed IRT for the measurement of early-stage explosive development and fireball expansion within a confined blast, with the temperature of the explosive fireball measured from its emitted radiance. The temperature measured by the IRT was corroborated against the temperature inferred from a pressure gauge measurement; both instruments measured the same temperature from the quasi-static pressure (QSP) point onwards. Before the QSP point, it is deduced that the IRT measures the average temperature of the fireball over a wide field-of-view (FOV), as opposed to that inferred from the singular shocks detected by the pressure gauge. Therefore, use of an IRT, in tandem with a pressure gauge, provides a potential invaluable measurement technique for the characterisation the early stages of a fireball as it develops and expands.

## 1. Introduction

Scientific characterisation of blast loads can be separated into three types of events: far-field blasts, near-field blasts, and confined blasts. Far-field blasts involve the target being sufficiently distant such that the loading is generated only by the impingement of the propagating air shock [1,2,3,4]. In contrast, near-field blasts involve the explosive charge being sufficiently close such that the explosive fireball impacts the target [1,5,6,7]. Finally, confined blasts involve detonation within a confined space, leading to the shock waves generated from the blast reflecting off its chamber walls and intermixing [5,8,9,10]. To elucidate more knowledge about what is happening within the early post-detonation stages, high quality experimental work is required. Whilst far-field loads are relatively well characterised, other blast load scenarios are not [5,7,11].

The scenario is particularly complex for confined explosions. Most high explosives comprise C-H-N-O compounds, which, when shocked, decompose rapidly in exothermic reactions, producing gaseous nitrogen, carbon dioxide and water. This sudden change of state and energy release results in a high temperature and pressure “fireball” of detonation products, which expands violently, compressing and displacing the atmosphere around it and generating shock waves. In many high explosives, there is insufficient oxygen in the compound to fully oxidise all the carbon. This means that the initial energy which goes into the fireball expansion and shock wave is formed from only a part of the full reaction energy, known as the *heat of detonation*. In a confined explosion, the shock waves will reflect from the confining waves and may return to the fireball, churning it up with the surrounding atmosphere. If there is sufficient oxygen and heat remaining, this may lead to a secondary combustion of the partially oxidised carbon, in a process called “afterburn”. In many explosives the *heat of afterburn energy release* is comparable to the heat of detonation. Over time, the shock waves in the confined atmosphere will lose their identity and the chamber will experience a more or less stable, increased pressure, called the “quasi-static pressure” or QSP. This will decay over time due to venting or heat losses to the confining walls.

The conventional genre of instrumentation used within blast measurements are pressure gauges; instruments which can provide a measurement of pressure from the shock waves produced by the blast. One common instrument used for the measurement of blasts is the piezo-resistive pressure transducer [1,12,13], with another type of instrumentation the Hopkinson Pressure Bar (HPB) [14,15]. Regardless of the type of pressure gauge used within a blast measurement, such instruments generally only provide a measure of pressure rather than temperature. Therefore, to extract temperature from these measurements, assumptions about the behaviour of the blast need to be made based upon ideal gas laws. For an accurate conversion of pressure to temperature using the ideal gas law, an assumption is made that these laws apply for the full duration of the blast. This may not necessarily be the case; ideal gas laws break down under high temperature and high pressure.

An alternative measurement approach is that of radiometry, an optical measurement technique that involves the detection of light from the infrared and visible parts of the electromagnetic spectrum. Such measurements are inherently non-contact; they do not need to be in contact with the target to measure the optical signal. Infrared radiation thermometers (IRTs) are one such instrument [16,17,18], which operate by measuring the radiated emission from the target, relating this to temperature in accordance with Planck’s Law [16,17,18]. When this radiance becomes incident upon the sensing element of the IRT, the sensor generates an output; a typical sensing element is a photodiode, from which photocurrent flows in proportion to the incident radiation. This photocurrent is amplified, converted to a voltage, and is calibrated to a temperature related to the magnitude of the radiated emission from the target in accordance with Planck’s Law.

Infrared radiation thermometers are used for a wide range of temperature measurement applications, and are prominent within industrial processing applications, for example, metals [19], glass [20] and petrochemicals [21]. They have also been used specifically for the measurement of temperature within applications featuring fast transient events. This facet makes them particularly attractive and suitable for the measurement of fireball temperature, which inherently involves fast transients. The IRTs have been used for many high-speed measurement applications, which we define as temperature transitions of faster than 10 μs for the purposes of this work. Example applications include: temperature measurement within dynamic compression [22], light emission signatures from ballistics [23], scanning mirror based IRTs [24], measurement of thin films and coatings [25], high-speed thermophysical property experiments [26] and thermal microscopes [27]. It should be noted that all these example applications involved the development of custom solutions for each specific temperature measurement application; commercial instruments are not available for such fast transient applications. 

It should be noted that IRTs are distinct from another optical measurement technique, that of imaging cameras—instruments that image the target over a larger area using an array of pixels. High-speed video (HSV) cameras are used within the characterisation of far-field blast loads [1,28], and enable both a spatial and temporal measurement of the fireball as it develops and expands. Whilst HSVs are suited for use within far-field blast load measurements, they are inherently positioned a distance from the target; they are not suited to confined blast or near-field measurements. Within such close measurement scenarios, the HSV would likely get damaged due to its proximity with the fireball.

In this work, we address the dearth of high-quality experimental data within the measurement of confined blast loads using a new approach to their characterisation: the development and application of a custom high-speed optical fibre based IRT. We present our high-speed IRT for the measurement of temperature within the early-stage explosive development and fireball expansion of a confined blast load. In turn, we demonstrate its ability to measure the temperature based on the emitted radiance of a fireball; confined blast loads have not previously been captured optically with such high temporal resolution. Comparisons are made with a temperature measurement inferred from a pressure measurement using a piezo-resistive pressure transducer-based pressure gauge. At the peak QSP point, both the IRT and pressure gauge measured a temperature of circa 1700 K, hence corroborating the use of the IRT within this application. We take this further by investigating the differences within the measurements from the two instruments prior to the stable QSP. We deduce that the IRT provides an average temperature of the fireball over a wider field-of-view (FOV), in contrast to the temperature inferred from the singular shock waves of the pressure gauge. Therefore, our IRT is shown to be an invaluable new tool for the characterisation of blast loads, particularly when used in tandem with pressure measurements. 

The organisation of this paper is as follows. Section 2 covers the Materials and Methods used within the paper, including: Section 2.1 Instrument Design, Section 2.2 Instrument Characterisation and Section 2.3 the Explosion Test Rig. The experimental results are presented within Section 3, including: Section 3.1 IRT characterisation and Section 3.2 the Blast Measurement. Section 4 provides further discussion of the results, and Section 5 provides some conclusions.

## 2. Materials and Methods

### 2.1. Instrument Design

**Figure 1:**
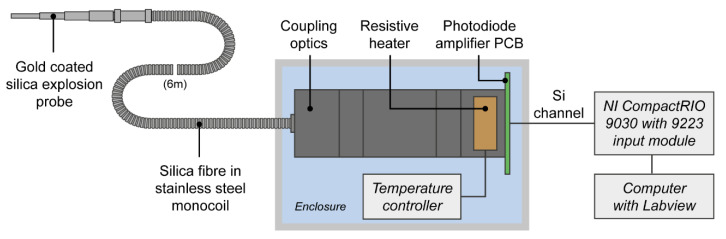
Overview of IRT and data acquisition system.

Figure 1 shows an overview of the IRT and its data acquisition system.

The IRT was built around a Hamamatsu K1713-09 Si-InGaAs two-colour photodiode, although only the Si channel was used within this work. The Si photodiode utilised a Texas Instruments OPA657 operational amplifier within a single-supply transimpedance amplifier (TIA) configuration for conversion and amplification of photocurrent to voltage. The transimpedance of the TIA was configured to enable the IRT to be calibrated for measurement of target temperatures between 1200 K and 2650 K, accounting for coupling losses within the optical fibre assembly. A transimpedance of 18 kΩ was chosen, with a feedback capacitor of 10 pF used for stabilisation and filtering. With the addition of a 1st order RC filter (R = 1 kΩ, C = 270 pF) added to the output of the TIA, the response time for the IRT was less than 1 μs. For reference, commercial IRTs typically have a response time down to 1 ms [29].

In order to capture the fast transient output from the IRT, a field programmable gate array (FPGA) based National Instruments (NI) CompactRIO 9030 controller incorporating an NI 9223 high speed, 4-channel 16-bit analogue input module was used. By utilising controlled sampling within Labview software, the instrument measured the IRT output voltage every 4 μs. The measurement data was stored within the CompactRio memory, prior to being extracted and converted to temperature. This data acquisition solution was chosen due to its scalability; it would be relatively straightforward to add additional IRT inputs whilst maintaining synchronous data acquisition at the same data acquisition rate.

**Figure 2:**
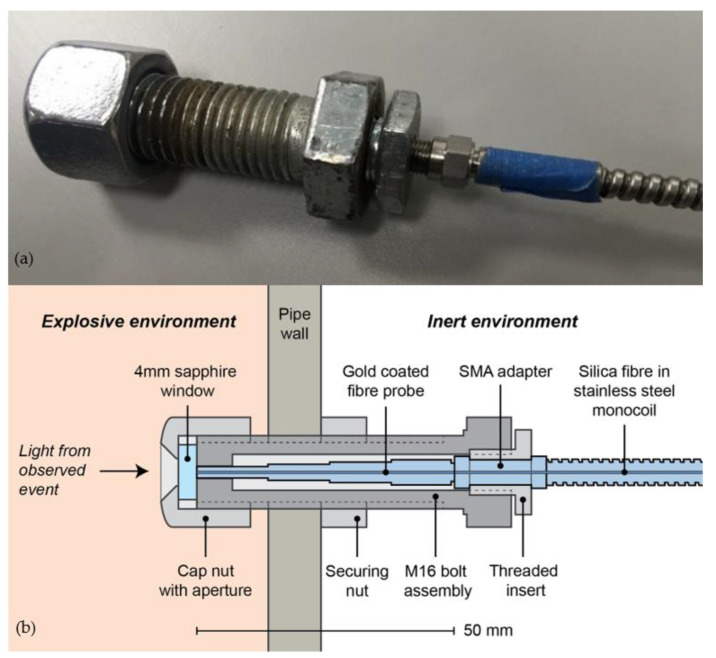
(**a**) Optical fibre probe within bolt for confined blast measurements. (**b**) Cross section of bolt used for glass measurement, indicating sapphire window.

The photodiode was optically coupled to a silica fibre-optic assembly using two N-BK7 Plano-Convex lenses in a symmetrical configuration within mechanical mounting. Ray trace modelling was performed in Zemax OpticStudio software (Zemax, Kirkland, WA, USA) to inform the design and dimensions, with the lenses positioned 30 mm apart; the photodiode and optical fibre were both positioned 46.2 mm from their respective lenses. No additional field or aperture stops were incorporated into the system. The optical fibre, encased within stainless steel monocoil sheathing to ensure robustness, was multi-mode, low hydroxyl ion (OH) type (operating wavelength range of 400–2200 nm), 6 m in length, 400 μm in diameter and had a numerical aperture (NA) of 0.22. A separate 50 mm long “patch cable” was coupled to the end of the main fibre for insertion into the explosion test rig. This “fibre probe”, with identical optical properties to the main cable, was gold coated to help ensure durability when exposed to high temperature blasts, and was mounted within a secure bolt, shown in Figure 2a, for attachment to the explosion test rig. The fibre probe was sighted through a detachable 4 mm thick sapphire window (operating wavelength range of 200–5500 nm), Figure 2b. This window was required to ensure the fibre surface was not coated in debris from the explosion, rendering it unusable for future tests. In contrast, the sapphire window could simply be removed and cleaned prior to subsequent tests. The fibre probe, including all its internal components, was attached to the main optical fibre prior to instrumentation calibration; this ensured that optical coupling losses were factored into the calibration.

### 2.2. Instrument Calibration

The central law governing blackbody radiation theory is Planck’s Law; IRTs are typically calibrated based upon this law or some variation of it [30]. It represents the relationship between the radiant emittance of a blackbody object as a function of temperature and wavelength, as shown in Equation (1).

(1) $$L\left(\lambda ,T\right)=\frac{c_{1}}{\lambda^{5}}\frac{1}{exp\left(\frac{c_{2}}{\lambda T}\right)-1}$$

where *L* represents the spectral radiance of a perfect blackbody emitter, whilst *c*_1_ and *c*_2_ represent the first and second radiation constants, respectively. Wavelength and temperature are represented by *λ* and *T*, respectively. The photocurrent generated from the photodiode, *I_ph_*, is proportional to *L* and, therefore, so is the IRT’s output voltage, *V*.

To calibrate the IRT in this work, a simple model using Equation (1), incorporating the wavelength dependent responsivity and optical system transmission, was first created to produce the “shape” of the IRT output as a function of target temperature. The IRT was subsequently sighted 20 mm from a target aperture placed in front of a Landcal R1500T furnace [31], with the output voltage corresponding to three calibration temperatures recorded. Using these three calibrations points, the calibration model was scaled to create a final calibration lookup table mapping *V* to *T*. To ensure traceability to ITS-90 [32] within the calibration, the temperature of the furnace was measured using an Ametek Land Cyclops C100L [33]. The nominal field-of-view (FOV) of the IRT was established using a series of decreasing diameter target apertures, which were placed in front of the furnace. In this work, we define the nominal FOV at the point which contains 98% of the target source’s radiant power based upon a paraxial image of the optical system’s field stop. A target aperture of 20 mm in diameter was used for the aforementioned instrument calibration; this represents the twice nominal FOV of the IRT. An assessment of the IRT’s size-of-source effect (SSE) [34], i.e., the impact of measuring a target which is larger in size than the IRT’s FOV, was also performed by increasing the target aperture diameter beyond this twice nominal point.

The root-mean-squared (RMS) noise of commercial IRTs, calculated by taking the standard deviation of the calibrated temperature measurement, have a typical specification of ±0.5 K [29]. This RMS noise specification helps to specify the minimum target temperature which the IRT is capable of measuring; ±0.5 K is deemed the maximum level of acceptable RMS noise within a measurement. However, commercial IRTs typically state this noise performance applies for measurements with a response time of 1 ms or greater [29]; a longer response, or integration, time allows for a greater degree of averaging within the measurement, enabling the RMS noise to be reduced. Therefore, to enable IRTs to operate at the speeds required to capture fast transient events, such as confined blasts, some compromise in the RMS noise performance, in terms of the minimum target temperature for an RMS noise of ±0.5 K, would be expected. In this work, the noise was assessed by recording the output voltage from the IRT with the CompactRio acquisition system at its raw 4 μs sampling rate over a 1 s period. This was calibrated to temperature, with the RMS noise, in K, calculated by taking the standard deviation of this calibrated data. By incorporating various degrees of centred averaging filters to the raw data, the noise equating to response times of 100 μs, 2 ms and 50 ms could also be assessed in the same way.

An 870 nm light emitting diode (LED) (Thorlabs LED870L) was used to verify the response time of the IRT and was positioned in front of the optical fibre. The LED was pulsed with a square wave generated from an Agilent 33210A function generator (frequency of 1 kHz, rise and fall times of 20 ns), with the resultant output voltage of the IRT circuit recorded using both a Keysight DSOX2002A oscilloscope and the CompactRio acquisition system. The final response time of the IRT is defined to be the duration between the 10% and 90% points upon the recorded square-wave step response.

The weakness of using IRTs for measuring temperature is the requirement to have a priori knowledge of the measurand emissivity [35]. According to blackbody theory [36], cavities enhance emissivity, with tubular and spherical cavities used as approximate blackbody calibration furnaces. The geometry of the enclosed blast chamber would suggest that the blast can be measured with the assumption that the measurand is a blackbody and emissivity can be ignored. 

### 2.3. Explosion Test Rig

**Figure 3:**
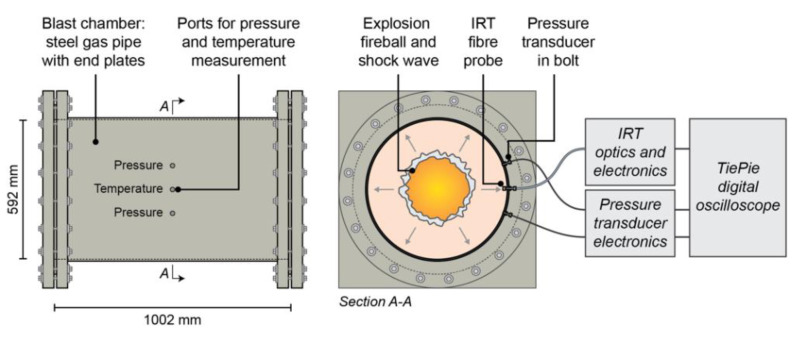
Test setup for blast measurement, incorporating IRT and pressure gauges instrumentation.

Figure 3 shows an overview of the measurement system used for blast measurements, showing both the IRT and pressure gauge instrumentation.

The pressure measurement was conducted using two Kulite piezo-resistive pressure sensors, each with a natural frequency of greater than 400 kHz; one 17 bar rated HKM-375 pressure gauge, and one 35 bar rated HEM-375M pressure gauge. These gauges were positioned off the main chamber in bolts, such as those described by Walter [37], hence ensuring their protection against the high temperature of the confined explosive event. This involved the pressure sensor being, as far as possible, isolated from direct contact with the detonation products; it was effectively mounted within a small air reservoir connected to the main blast chamber. Prior to conducting any tests, each gauge was first validated using a static pressure pump; a known pressure was applied to the gauge to check that it resulted in a rise in output voltage. An assessment of whether this voltage rise coincided with the calibration factors provided by the manufacturer, in units of mV/BAR, was performed.

The output voltages from the pressure gauges were captured using a Tie-Pie Handyscope HS6 data logging oscilloscope. Such an approach enables data to be recorded at either 14- or 16-bit resolution, depending on the desired measurement precision or overall required measurement duration. The oscilloscopes were set to trigger upon detection of a voltage larger than the background noise, with data logging commencing at time zero when the gauge experiences a rise in pressure within the chamber, and a concomitant rise in voltage. Although the oscilloscope constantly measured the voltage from the pressure gauges, data logging only commenced upon detection of a voltage rise. A 10% pre-trigger was applied to the system to ensure all the credible information from the test was recorded.

The captured data was converted to pressure using the gauge calibration factor and an average of the baseline data, as per Equation (2), where *P* is pressure, *V* is voltage, *V_o_* is the pre-event datum voltage and *CalF* is the calibration factor of the gauge.

(2) $$P=\left(V-V_{o}\right)\times CalF$$

In order to extract temperature from the pressure measurement, the ideal gas law is assumed, as per Equation (3), where *P* is absolute pressure, *V* is volume, *n* is the amount of substance, *R* is the gas constant (8.314 J/(mol.K) and *T* is the temperature.

(3) PV=nRT

In this work, a blast load of 50 g of a plastic-based explosive known as Plastic Explosive No. 4 (PE-4) [38] was detonated within an air environment. The volume of the chamber was 275 L, whilst the value of *n* used was calculated as follows: (i) the number of moles of air in the chamber prior to the test was determined via Equation (3), using measurements of the ambient atmospheric pressure and temperature. (ii) The number of moles of gas produced by the complete reaction of the explosive charge was determined and added to this value. (iii) Finally, the number of moles of oxygen from the air in the chamber required to facilitate the complete reaction of the explosive charge was deducted. Via this approach, *n* was found to be 11.5 mol pre-test and 13.0 mol after the detonation. 

## 3. Results

### 3.1. IRT Characterisation

**Figure 4:**
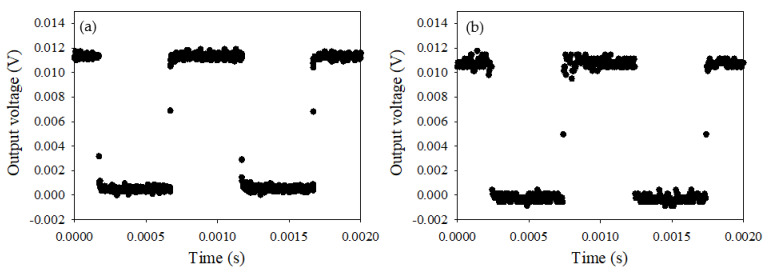
Response time assessment of the IRT with a 870 nm LED pulsed with a 1 kHz square wave, measured by (**a**) the oscilloscope (**b**) the CompactRio acquisition system.

To radiometrically validate the use of the IRT for the measurement of temperature within confined blasts, the IRT was calibrated and characterised using the methods described in Section 2.2. The response time of the IRT to an LED pulsed with a square wave, with its output connected to an oscilloscope, is shown in Figure 4a. Rise and fall times were found to be less than 1 μs, indicating that the response time of the analogue electronics was sufficiently faster than the 4 μs logging rate of the digital CompactRio acquisition system. Figure 4b shows the equivalent measurement, with the output of the IRT this time connected to the CompactRio acquisition system.

**Figure 5:**
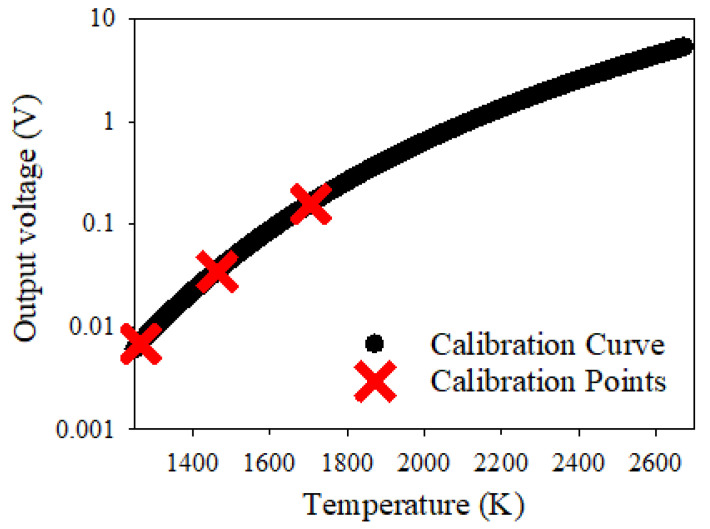
Output voltage as a function of temperature for calibrated IRT. Red crosses represent the calibration points at temperatures of 1265, 1463 and 1705 K.

Figure 5 shows the output voltage of the IRT as a function of blackbody temperature, showing the three calibration points along with the resultant calibration curve mapping output voltage to temperature. The calibration was carried out at temperatures of 1265 K, 1463 K and 1705 K, producing a calibration curve between 1200 K and 2650 K; the IRT was able to measure temperatures of the confined blast of up to this maximum temperature.

**Figure 6:**
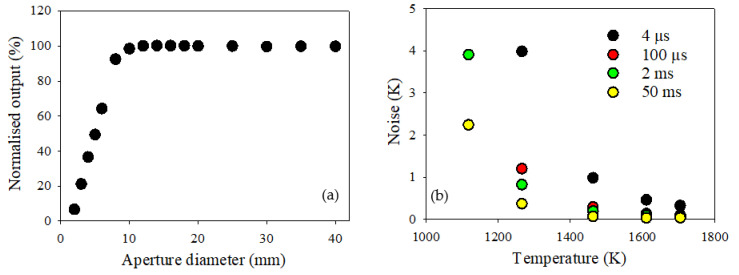
(**a**) FOV and SSE for the IRT and (**b**) RMS noise as a function of target temperature for integration times of 4 μs, 100 μs, 2 ms and 50 ms.

The FOV of the IRT was measured, as shown in Figure 6a, with the nominal FOV found to be approximately 2:1. This means that the diameter of the target area in which the IRT is sighted upon is half the distance at which the IRT is positioned away from the target. It can be assumed that, if the fireball is larger than twice this nominal FOV, the calibration to temperature is valid; the fireball diameter needs to have grown to equal the distance at which the IRT is positioned away from the target. The SSE was found to be minimal, indicating that further increase in fireball size beyond this twice nominal FOV has negligible impact upon the temperature measurement; the temperature of the fireball will continue to be accurately measured.

The RMS noise as a function of furnace temperature for the IRT is shown in Figure 6b. The ±0.5 K noise specification was found at a furnace temperature of approximately 1600 K for the raw 4 μs response time of the IRT. Whilst this minimum measurement temperature is relatively high compared to commercial IRTs, a response time of 4 μs is significantly faster, further integration of the measurement data leads to a significant improvement in the RMS noise performance. For example, this minimum temperature is reduced to values of less than 1400 K, 1350 K and 1250 K for integration times of 100 μs, 2 ms and 50 ms, respectively, as shown in Figure 6b. However, given that the typical temperatures expected from a blast event such as those investigated in this work are higher than the ±0.5 K noise specification temperature of 1600 K, the noise within the blast measurements is low.

### 3.2. Blast Measurement

**Figure 7:**
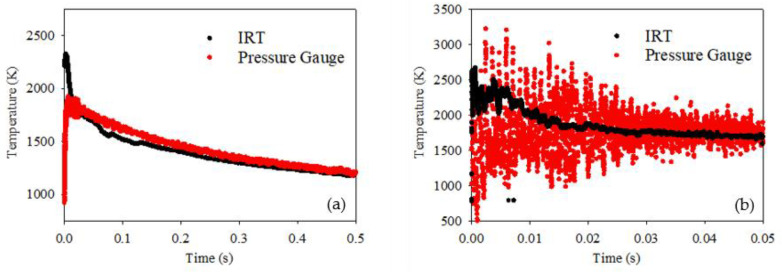
Temperature measurement of the confined blast for the (**a**) first 500 ms of the fireball, with 2 ms filter, and (**b**) first 50 ms of the fireball, without 2 ms filter.

An explosive charge of 50 g PE-4 was detonated within an air atmosphere using the setup shown in Figure 3. The raw data captured from the respective gauges was converted to temperature, using the aforementioned calibration techniques, as shown in Figure 7. Note, Figure 7a shows the first 500 ms of the blast, with a 2 ms centred average filter applied to both sets of measurements. In contrast, Figure 7b shows the first 50 ms of the blast, with the 2 ms filter removed.

The temperature inferred from the pressure measurement follows the trend expected from such a blast load; the temperature rises due to a build-up in pressure, with the fireball filling the chamber. A brief plateau in this inferred temperature reading is found at around 1800 K; this is the temperature inferred when peak QSP is reached, which occurs between 10 ms and 20 ms. The temperature at QSP is consistent with that which would be expected for a load of this type, corresponding to a pressure of circa 600–700 kPa. There is a subsequent fall in the maximum QSP, with the temperature concomitantly decreasing due to thermal losses to the chamber walls.

The trend observed by the IRT temperature measurement is similar: increase in temperature with the initial onset of the fireball, followed by a drop-off following the decrease in pressure inside the chamber. However, during the first 10 ms of the blast, the measurements from the two instruments differ. Whilst the pressure gauge indicates that the pressure within the chamber is still increasing, the IRT measures a temperature several 100 K higher, as shown for the filtered data within Figure 7a. Observation of the non-filtered IRT measurement within Figure 7b suggests a peak temperature reading of circa 2600 K, staying above 1800 K over the first 10 ms, before levelling off from 10 ms to 20 ms at QSP. For the remainder of the blast’s duration beyond QSP, the IRT measurement follows that of the pressure gauge measurement; a steady drop-off in temperature following the reduction in pressure within the chamber. This strongly implies that the assumption of ideal gas behaviour is reasonable over the medium term, but the early post-detonation discrepancies demand further attention. 

Over the first 10 ms of the blast, the IRT measures a temperature of over 1800 K, whilst the pressure gauge measures multiple, spaced peaks and troughs. These peaks and troughs are associated with the multiple reflections of the confined shock waves. It is possible that the IRT is essentially providing an average temperature reading over its entire FOV, whilst the pressure gauge is measuring individual shock waves. Whilst these peaks within the pressure gauge temperature measurement may be of a similar, albeit noisier, order to the IRT, the filtered temperature measurement is lower. During the first 10 ms, the pressure gauge is effectively only measuring individual spaced shocks; once averaged, the final measured temperature will be lower. Alternatively, it may be that the IRT is directly measuring the flame temperatures of the secondary afterburn reactions as the shock waves churn up the still fuel-rich fireball with the oxygen in the chamber atmosphere. These temperatures would not be proportional to the chamber pressure, as they are due to a chemical reaction rather than a compression of the gas, and hence would not be picked up by the temperature inferred from the pressure gauge. Work is continuing to determine the cause of this early-stage discrepancy.

The nominal FOV of the IRT was measured to be 2:1, as shown in Figure 6a, with the measurement deemed valid at twice this nominal FOV. Therefore, the diameter of the fireball needs to be equivalent to the distance that the IRT is away from the fireball. Based upon the charge used and the dimensions of the setup, the fireball was estimated to grow to a size of approximately 600 mm after 0.18 ms from the point of detonation. At this point, the FOV of the IRT is filled, and quantitative temperature measurements deemed valid. Given that this occurs at an early point within the lifetime of the fireball, all the results shown in Figure 7 are deemed valid.

## 4. Discussion

The correlation in the temperatures measured by the two instruments at and beyond QSP validates the use of the IRT for the measurement of temperature within a confined blast. This demonstrates it is a useful tool for the measurement of such blasts, especially due to the fast response time and lower noise performance offered by its 4 μs acquisition speed in comparison to the pressure measurement. However, the inherent nature of its optical measurement, in contrast to a pressure measurement, provides additional information about what may be happening during the very early stages of a fireball. The IRT measurement within Figure 7 suggests that the temperature of the fireball is higher over the first 10 ms of the blast compared to the filtered pressure gauge measurement. The IRT effectively measures an average temperature reading over the surface of the fireball, in contrast to the individual shock waves detected by the pressure gauge. By combining this approach to measuring the surface temperature of the fireball, along with the lower level of noise within the data, a more accurate measure of temperature can be deduced within the early stages of the fireball by using the IRT.

**Figure 8:**
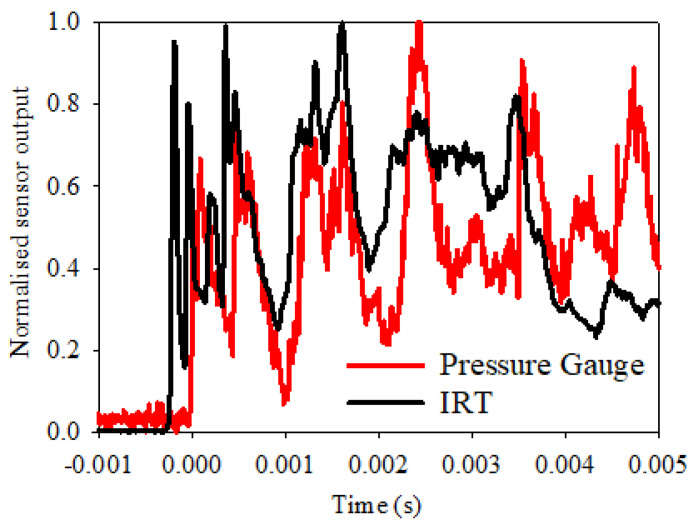
Comparison of the normalised sensor outputs of the confined blast for the first 5 ms of the fireball with both sensors connected to the same data logger.

In order to perform an accurate temporal investigation into the very early stages of the fireball, it is important for the IRT and pressure gauge outputs to be accurately synched together; any discrepancy in the temporal synchronisation will lead to inaccurate conclusions when such short timeframes are concerned. To enable this, the IRT output was connected to the Tie-Pie Handyscope along with the pressure gauge, and another explosive charge of 50 g PE-4 within air was detonated. Figure 8 shows the normalised outputs of the two sensors over the first 5 ms of the blast, with no centred average filter added to either set of results. For reference, 0 ms on the time-axis represents the initial point at which the pressure gauge registered an initial increase of pressure within the chamber, in according with the configuration of the setup described in Section 2.3.

A sharp increase in signal is observed from the IRT reading, with the pressure gauge response rising approximately 200 μs later; this trend is repeated over the first 5 ms of the blast. Whilst this might suggest a rise in temperature before a rise in pressure, the reality is likely more nuanced. It is likely that the IRT registers data sooner due to a combination of the IRT measuring over the aforementioned larger FOV, compared to individual shocks detected by the pressure gauge, and the fact that light travels faster than shock waves. The IRT essentially detects the presence of the fireball before the pressure gauge does, therefore providing a sooner measure of temperature. This ultimately makes it possible for the IRT to gather more information about what might be happening in the early stages of a fireball as it develops and expands. It would also be feasible, with the use of further photodiode circuit optimisation combined with a faster data acquisition system, for an even faster IRT temperature measurement. This would enable sub-microsecond temperature information within the fireball to be acquired, enabling further exploration of what may be happening within the early stages of a fireball as it develops and expands, as well as at other stages within its lifetime.

## 5. Conclusions

We have demonstrated the use of a high-speed IRT for the optical measurement of temperature within a confined blast. The IRT was radiometrically characterised using a pulsed LED and blackbody furnace, before being demonstrated for the measurement of a confined blast load. The IRT’s temperature measurement was compared against a measurement of temperature inferred from a piezo-resistive pressure transducer, with both instruments producing the same temperature reading from the QSP point onwards. The differences between the two readings before the QSP point were analysed, with results suggesting that the IRT provides an average temperature measurement of the fireball over a wider FOV, in contrast to the temperature inferred from the singular shock waves of the pressure gauge. Therefore, by using the IRT, especially in tandem with a pressure gauge, we can provide a more accurate, high-speed temporal investigation into what may be happening in terms of temperature within the very early stages of a fireball as it develops and expands. Our work directly addresses the dearth of high-quality experimental data within the measurement of confined blast loads, by demonstrating a new, high-speed optical approach to their characterisation.

## Data Availability

All relevant data are shown in the paper or could be recreated by following the methodology in the paper.
